# Influence of Endogenous Cardiac Glycosides, Digoxin, and Marinobufagenin in the Physiology of Epithelial Cells

**DOI:** 10.1155/2019/8646787

**Published:** 2019-12-30

**Authors:** Alejandro Ogazon del Toro, Lidia Jimenez, Lorena Hinojosa, Jacqueline Martínez-Rendón, Aida Castillo, Marcelino Cereijido, Arturo Ponce

**Affiliations:** Department of Physiology, Biophysics and Neurosciences, CINVESTAV-IPN, CDMX, C.P. 07360, Mexico

## Abstract

Cardiac glycosides are a group of compounds widely known for their action in cardiac tissue, some of which have been found to be endogenously produced (ECG). We have previously studied the effect of ouabain, an endogenous cardiac glycoside, on the physiology of epithelial cells, and we have shown that in concentrations in the nanomolar range, it affects key properties of epithelial cells, such as tight junction, apical basolateral polarization, gap junctional intercellular communication (GJIC), and adherent junctions. In this work, we study the influence of digoxin and marinobufagenin, two other endogenously expressed cardiac glycosides, on GJIC as well as the degree of transepithelial tightness due to tight junction integrity (TJ). We evaluated GJIC by dye transfer assays and tight junction integrity by transepithelial electrical resistance (TER) measurements, as well as immunohistochemistry and western blot assays of expression of claudins 2 and 4. We found that both digoxin and marinobufagenin improve GJIC and significantly enhance the tightness of the tight junctions, as evaluated from TER measurements. Immunofluorescence assays show that both compounds promote enhanced basolateral localization of claudin-4 but not claudin 2, while densitometric analysis of western blot assays indicate a significantly increased expression of claudin 4. These changes, induced by digoxin and marinobufagenin on GJIC and TER, were not observed on MDCK-R, a modified MDCK cell line that has a genetically induced insensitive *α*1 subunit, indicating that Na-K-ATPase acts as a receptor mediating the actions of both ECG. Plus, the fact that the effect of both cardiac glycosides was suppressed by incubation with PP2, an inhibitor of c-Src kinase, PD98059, an inhibitor of mitogen extracellular kinase-1 and Y-27632, a selective inhibitor of ROCK, and a Rho-associated protein kinase, indicate altogether that the signaling pathways involved include c-Src and ERK1/2, as well as Rho-ROCK. These results widen and strengthen our general hypothesis that a very important physiological role of ECG is the control of the epithelial phenotype and the regulation of cell-cell contacts.

## 1. Introduction

Cardiac steroids (CS) are a group of diverse compounds derived from plant and animal sources, with similar chemical properties, that have long been used to increase cardiac contractile force in patients with congestive heart failure and cardiac arrhythmias. They all contain a structure consisting of 17 carbon atoms arranged in 4 cyclic carbon rings, known as sterane moiety, and an unsaturated lactone ring at the C-17 position. Unlike sex hormones, mineralocorticoids and glycocorticoids, which are all transconnected, cardiac steroids show an A/B and C/D cis-conformation [1, 2]. A subset of cardiac steroids, known as cardiac glycosides (CG), also contain one or more glycosidic residues at the C-3 position. Depending of the structure of the C17-lactone substituent, cardiac glycosides are further classified as cardenolides or bufadienolides. Cardenolides have a five-membered lactone (butenolide) substituent, whereas bufadienolides have a six-membered unsaturated lactone (*α*-pyrone) [3].

More than a hundred cardiac glycosides have been identified as secondary metabolites in plants, including *Strophanthus* (that produces ouabain), *Digitalis lanata* and *Digitalis purpurea* (producing digoxin and digitoxin), *Scilla maritima* (producing proscillaridin A), and *Nerium oleander* (that produces oleandrin and oleandrigenin) [4]. Some species of amphibians and reptiles also produce cardiac glucosides. Several bufadienolides, including marinobufagin, proscillaridin, and bufalin, are isolated from the skin toads of genus *Bufo* [5]. These substances give the plants or animals that produce them, toxic or even poisonous properties; for this reason, they have been used since long ago for hunting or warfare and in controlled doses for various medicinal or therapeutic purposes, among which its use in heart-related problems stands out. They are used also as pesticides, emetics, diuretics, and even as tinctures [6, 7].

Some CG, including ouabain, digoxin, and digitonin, have been used as cardiac inotropic agents for almost 200 years; however, due to their narrow therapeutic index, the CG have been gradually replaced by other medications and presently are almost discontinued for this purpose [8]. Nonetheless, a fact that has given a renewed interest, on the study of these compounds, is the finding that apart from their effect on heart and hypertension, they influence an interesting variety of physiological and pathological processes, such as cell adhesion [9], growth, apoptosis, motility, and differentiation [10–12]. Among these, the ability to induce impairment of cell proliferation or activation of cell death by apoptosis or autophagy has led to consider CG as promising new therapeutic tools against cancer [13–16]. Cardiac glycosides have also been found to decrease inflammatory symptoms [17]. The mechanism by which cardiac glycosides exert an inotropic effect on cardiac muscle, is known since several decades. These compounds inhibit the pumping activity of the Na-K-ATPase pump, raising intracellular Na+, which in turn inhibits the function of the Na+/Ca2+ exchanger, reducing the exchange of extracellular sodium with intracellular calcium, bringing as a consequence, an increase in intracellular calcium [18]. A second hypothesis, about the way that cardiac glycosides interact with Na-K-ATPase, has been described more recently. It indicates there exists a subpopulation of Na-K-ATPase, located in caveolae that does not function as a pump, but rather as a receptor that upon binding of cardiac glycosides activates one or more signaling pathways to produce a variety of changes on the physiology or even the genetic expression of cells [19, 20]. The binding of cardiac glycosides to Na-K-ATPase activates the Src/epidermal growth factor receptor complex to initiate multiple signal pathways, which include PLC/IP3/CICR, PI3K, reactive oxygen species (ROS), PLC/DG/PKC/Raf/MEK/ERK1/2, and Ras/Raf/MEK/ERK1/2 pathways [21].

A second fact that has given renewed interest to the study of cardiac glycosides is the finding that some of these compounds are produced endogenously by some mammalian species, including humans. Endogenous Cardiac Steroids (ECS) include ouabain, digoxin, marinobufagenin, and proscillarin A among a few others [22–28]. In the last two decades, it has been described that these compounds are found in almost all mammalian tissues, including blood plasma and urine. Their levels, which are in the pico to nanomolar range increase during pregnancy, physical exercise, or in a high salt diet [29, 30]. These findings have led to consider endogenous cardiac glycosides as a new class of steroid hormones and has prompted interest in their physiological role [31–33].

In the past years, we have focused on studying how ouabain influences the physiology of epithelia using Madin–Darby Canine Kidney (MDCK), a dog-derived kidney tubule epithelial cell line that has been extensively used as an epithelial model [33, 34]. We have shown that ouabain (10 nM), produces remarkable changes in some crucial aspects of the morphology as well as the physiology of epithelia, most of them related to contacts and cooperation between neighboring cells [35]. Thus, we have demonstrated that ouabain influences Tight Junctions (TJ), as reflected by a significant increase in the Transepithelial Electrical Resistance (TER) and to an increased expression of claudins 2 and 4 [36]. It also induces, within minutes, Gap Junctional Intercellular Communication (GJIC) by promoting relocation of connexins 32 and 43 [37, 38]. Ouabain 10 nM upregulates Adherens Junctions, enhancing the cellular content of E-cadherin, *β*-catenin, and *γ*-catenin [39]. The same treatment influences the apical/basolateral polarity, as reflected in the acceleration induced by ouabain in the ciliogenesis of the cells that develop a mature monolayer [40]. More recently, it has been shown that ouabain 10 nM accelerates collective cell migration of MDCK in wound healing assays [41]. In addition to demonstrating the influence of ouabain in the processes already described, we have shown that Na-K-ATPase is the primary receptor that mediates a signaling cascade involving c-Src and ERK1/2 [42].

Given our previous results, in this work, we analyzed whether digoxin and marinofufagenin, another ECS, would produce a similar effect in these two properties of epithelial cells. We also tested if, like with ouabain, Na-K-ATPase is the primary receptor that mediates such responses and whether c-Src, ERK1/2, and Rho/ROCK participate in the signaling pathways involved.

## 2. Methods

### 2.1. Cell Culture

Wild type MDCK cell line was obtained from the American Type Culture Collection and culture according to recommendations given; MDCK-R, a subclone highly resistant to ouabain, was kindly provided by Dr. Louvard (Pasteur institute). For production and maintenance, cells (both MDCK-W and MDCK-R) were grown in a 5% CO_2_ atmosphere at 36.5°C in Dulbecco's Modified Eagle Medium (DMEM; Gibco), supplemented with penicillin-streptomycin 10,000 U/*μ*g/ml (Cat. 15140122, Thermo Fisher Scientific), and 10% fetal bovine serum (Gibco). This medium is hereafter referred to as CDMEM. For all experimental assays, cells were harvested with trypsin (In Vitro Technologies, Australia), seeded at confluency (1-2 × 10^4^ cells/cm^2^), and kept in CDMEM during 24 hours (to allow monolayers to mature) and then incubated for additional 24 hours in a starvation media (which has the same composition as CDMEM but with fetal bovine serum reduced to 1%) before addition of cardiac glycoside treatments.

### 2.2. Measurement of Gap Junctional Intercellular Communication by Dye Transfer Assays

Glass micropipettes, with a tip resistance of 5–10 MΩ, were backfilled with a solution containing 120 mM KCl, 5 mM NaCl, 1 mM MgCl2, 5 mM HEPES (pH 7.4), and Lucifer yellow (1%). After filling up, pipettes were attached to a holder device and mounted to a micromanipulator (PCS-750; Burleigh Instruments) for cell impalement. Coverslips on which mature MDCK monolayers had been grown, were placed in a chamber containing PBS plus Ca^2+^ (1.8 mM) solution, at room temperature. Then, the chamber was mounted on the stage of an inverted, epifluorescence-equipped microscope (Diaphot 300; Nikon) to monitor impalement and injection. Cells, which were randomly chosen from among those constituting the monolayer, were impaled and injected one at a time, with Lucifer yellow, using a pneumatically driven microinjecting device (IM300; Narishige). After about 30 to 50 cells injected, the coverslips were rinsed with PBS and fixed by dipping into 4% paraformaldehyde, then rinsed (3x) with PBS, and mounted using VECTASHIELD® (H-1000; Vector Laboratories, Burlingame CA, USA). Some coverslips were dipped into PBS-Propidium iodide (0.1%) to stain nuclei and rinsed before mounting. Eight-bit images of the fluorescent cells were acquired at room temperature using a Zeiss M200 inverted microscope equipped with a Plan-NeoFluar 63x N.A. 1.25 objective lens, an AxioCam MRm camera, and Axovision 4.8 software. The captured images were imported into ImageJ software (release 2.8, NIH, Bethesda, MD, USA) to adjust the brightness and the contrast and GIMP (release 2.8.10, NIH) to compose the figures.

### 2.3. Measurement of Transepithelial Electrical Resistance (TER)

Monolayers were grown directly on Transwell® culture inserts (Costar Corning, Ref. 3415) by seeding MDCK cells at a density of 2 × 10^4^ cells/cm^2^ and left to mature for a 24 hours and then starved for additional 24 hours before addition of treatments to media. TER was measured before at 0, 24, 48, and 72 hours after addition of treatments, using an EVOM (Epithelial Voltohmmeter; World Precision Instruments). Measurements are expressed as ohms per square centimeter (Ω·cm^2^).

### 2.4. Immunofluorescence Assays

Coverslips, on which MDCK monolayers had been grown, were washed three times with ice-cold PBS-Ca^2+^, then fixed and permeabilized by dipping into methanol for 8 min at −20°C, then rinsed 3X with PBS, and blocked for 1 h with 0.5% BSA in PBS at room temperature. To stain claudin-2, samples were first incubated with a polyclonal anti-claudin-2 antibody (Cat #51–6100, Invitrogen, 1 : 400, overnight at 4°C), rinsed 3X and incubated (1 hour at room temperature) with a biotin-conjugated secondary antibody (Goat anti-Rabbit IgG (H + L), Cat #31822, Invitrogen, 1 : 2000), and rinsed 3X and finally incubated with Streptavidin-FITC (1 hour, 1 : 3000). Staining of claudin-4 was performed by overnight incubation of samples with a monoclonal anti-claudin-4 antibody (Cat. no. 32-9400, Invitrogen, 1 : 400) as primary and FITC-conjugated Goat Anti-Mouse IgG (H + L) (Cat. no 62-6511, Invitrogen, 1 : 2000) as secondary. Nuclei of cells were counterstained in blue by dipping the coverslips in PBS containing 5 mg/ml 1% DAPI (10236276001 ROCHE, Sigma-Aldrich, 20 min) The coverslips were mounted using Vectashield mounting medium (Cat. no H-1000, Vector Laboratories) and examined by confocal microscopy (SP8, equipped with a Plan-NeoFluar 63x NA 1.4 objective, Leica Microsystems, Wetzlar, Ger).

### 2.5. Western Blotting

#### 2.5.1. Production and Processing of Samples

Monolayers were grown on 35 mm Petri dishes, then untreated (CONTROL) or treated with either 100 nM Digoxin (DGX) or 100 nM Marinofufagenin (MBG) for 48 hours, rinsed twice in cold PBS and covered with 200 *μ*l of lysis solution (20 mM Tris (pH 7.0), 2 mM EGTA, 5 mM EDTA, 30 mM sodium fluoride, 40 mM *β*-glycerophosphate (pH 7.2), 1 mM sodium orthovanadate, 3 mM benzamidine, 0.5% Nonidet P-40, and cOmplete™, protease inhibitor mixture (Roche Applied Science, Germany)), and then frozen at−20°C for 15 minutes. Lysate samples were scraped with a policeman and then centrifuged for 15 minutes at 4°C, 14000 RPM. Supernatants were recovered and combined with Laemli sample buffer (1 : 1), aliquoted, and frozen at−70°C. Total protein was quantified using the bicinchoninic acid (BCA, Pierce) method, according to kit supplier instructions.

#### 2.5.2. Western Blotting

A volume containing 10 *μ*g of protein from each sample was loaded on 15% acrylamide gels, which were run at 100 V for 1 hour and 20 minutes. Proteins were then transferred to PVDF membranes activated in cold methanol. Semiwet transference was performed at 300 mA for 1 hour. Membranes were blocked overnight with a solution of 5% albúmin dissolved in TBS-T; afterwards, membranes were incubated with primary anti-claudin-4 antibody for 1 hour (1 : 500, diluted in 5% BSA), rinsed 10 times in TBS-T, then incubated with secondary anti-mouse antibody, and rinsed 10 times with TBS. Membrane stains were detected with the enhanced chemiluminescence kit (Amersham Biosciences) and visualized on Kodak X-Omat film. After that, membranes were stripped off by dipping into a solution of 2% dithionite-TBS. Once striped, membranes were probed for actin (primary anti-actin antibody 1 : 5000, 1 hour incubation, and secondary anti-rabbit antibody 1 : 5000). Band densities were measured on ImageJ software, and the density of each claudin-4 band was divided between the density of each corresponding actin band, and then the density of the DGX and MBG bands were compared to control. Anti-claudin-4 antibody and HRP-conjugated anti-rabbit and -mouse IgG were purchased from Zymed Laboratories.

### 2.6. Chemicals

Digoxin (Sigma-Aldrich 20830-75-5) and marinobufagenin (Cayman Chemical 470-42-8) stocks were prepared in DMSO and absolute ethanol, respectively. Subsequent dilutions were made with PBS without calcium. Lucifer Yellow was obtained from Sigma-Aldrich (67764-47-5) In the corresponding assays, cells were exposed to 10 *μ*M PP2, an inhibitor of c-Src kinase (MEK-1; 513000; Merk Millipore, Darmstadt GE) and 25 *μ*M PD98059, an inhibitor of mitogen extracellular kinase-1 (529573; Merk Millipore). Y-27632 (Merck KGaA, Cat 688000, Darmstadt, Germany) was prepared as a 10 mM stock in water and used at a concentration of 1 *μ*M.

### 2.7. Statistical Analyses

Statistical tests were performed with the analysis module of Sigmaplot or EXCEL software (Microsoft). The results are expressed as the mean ± standard error. Statistical significance was estimated via ONE-WAY analysis of variance (ANOVA) followed by Bonferroni's multiple comparison or Student's *t*-test and was denoted as follows: ^*∗*^*P* < 0.05, ^*∗∗*^*P* < 0.005, and ^*∗∗∗*^*P* < 0.001, and *n* is the number of observations obtained from at least 3 independent experiments.

## 3. Results

### 3.1. Effect of Digoxin and Marinobufagenin on Gap Junctional Intercellular Communication of Epithelial Cells

**Figure 1:**
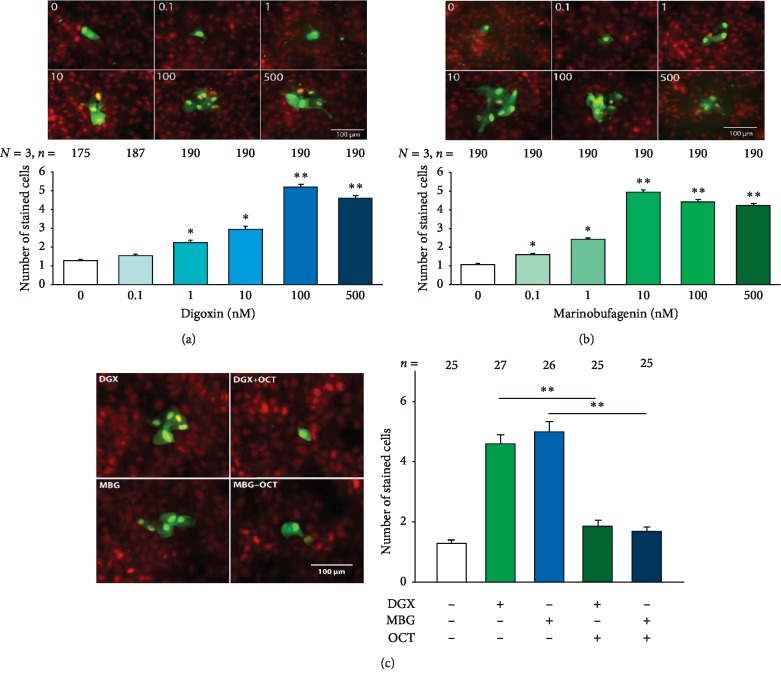
Digoxin and marinobufagenin enhance Gap junctional intercellular communication (GJIC) in MDCK cells in mature monolayers. (a) Effect of digoxin. (b) Effect of marinobufagenin on the CIGJ. Both figures show, in the upper part, a set of representative images, one for each one of the different concentrations tested. Multiple cell staining results from injection of a cell with Lucifer Yellow (green) which then diffuses to their neighbors through gap junction. Samples were counterstained with propidium iodide (red) to reveal the presence of nuclei in noninjected and injected cells. The histogram, in the bottom, part compares the average number of cells stained by injection, of a single cell, with Lucifer Yellow. (c) In the left part, a set of representative dye injection trials made in monolayers treated with either digoxin (100 nM) or marinobufagenin (10 nM) in the absence or presence of 1 mM octanol. The right part compares the average number of stained cells in the presence or absence of octanol. Numbers above each bar indicate the number of trials. The asterisks indicate a statistically significant difference compared to the 0 nM group (Dunn's method); ^*∗*^*p* ≤ 0.05, ^*∗∗*^*p* ≤ 0.001.

To determine if either digoxin or marinobufagenin, or both, influence GJCI, we made dye transfer assays (as described in methods) in MDCK cells from mature monolayers grown on coverslips, treated, with either digoxin or marinobufagenin, at concentrations of 0, 0.1, 1, 10, 100, or 500 nM for 1 hour. In each coverslip, multiple repeats were made, and each repeat consisted of the injection of Lucifer yellow into a single cell, which was able to diffuse to the neighboring cells if there was GJIC. Then, the average number of cells stained per trial was considered as a criterion to estimate the degree of GJIC. Therefore, for each experimental treatment, we compared the average number of stained cells versus the corresponding value obtained from untreated (control) cells.  Figure 1(a) shows the results with digoxin, while Figure 1(b) shows the results with marinobufagenin, and in both cases (A and B) in the upper part representative images of cells stained by transfer of Lucifer Yellow are shown. The lower part of each figure (either 1A or 1B) shows a bar chart, comparing the average number of cells stained at the different concentrations assayed as well as those untreated (0 nM). Statistical analysis indicates that both digoxin and marinobufagenin induce CIGJ (Kruskal–Wallis One-Way Analysis of Variance on Ranks, *P* < 0.001), although with different sensitivity. As observed in the graphs, the average number of stained cells treated with marinobufagenin starts to be significantly higher than that from control (Dunn's Method, *p* ≤ 0.05), from 0.1 nM, while with digoxin a significant difference is observed from 1 nM. In both cases, the average number of stained cells reaches a maximum and then decreases as the concentration of cardiotonics increases. The peak with marinobufagenin is at 10 nM, while with digoxin it is at 100 nM.

To verify that diffusion of dye from the injected cell to the neighbors is through gap junctions, we made a second round of dye transfer assays to compare the average number of stained cells obtained in the presence or absence of octanol (1 mM), a compound known to uncouple gap junctions [43]. As shown in Figure 1(c), the presence of octanol significantly reduced the average number of stained cells induced by digoxin or marinobufagenin (*p* < 0.005, Duncan Method) which indicates that the cells that are neighbors of the injected cells are stained by diffusion of yellow Lucifer through gap junctions. Therefore, from these experiments, we concluded that both digoxin and marinobufagenin in concentrations in the nanomolar range, induce enhancement of GJIC in epithelial cells.

### 3.2. Effect of Digoxin and Marinobufagenin on Tight Junctions

**Figure 2:**
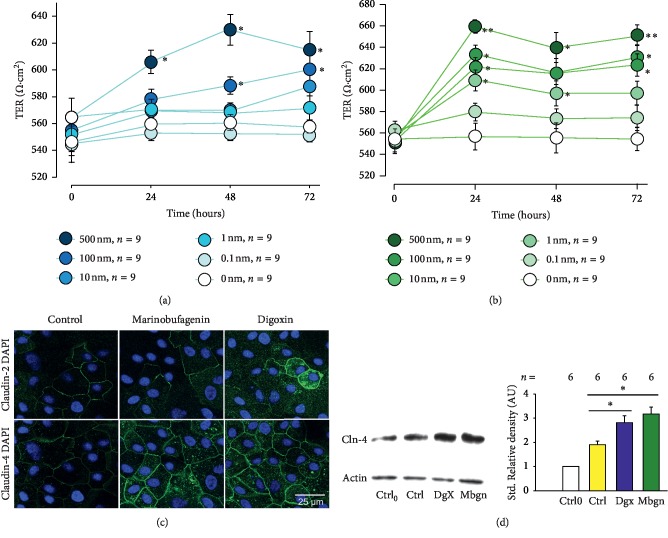
Digoxin and marinobufagenin, in a nanomolar range, promote a statistically significant increase in transepithelial electrical resistance (TER) in monolayers of MDCK cells. (a, b) Relationship of TER versus time of digoxin and marinobufagenin, corresponding to the different concentrations tested, including control (0 nM). Each dot represents the average ± SE value of TER (Ohms·cm^2^) of nine repeats of three independent assays. The asterisks indicate a statistically significant difference obtained after comparison of the value at any concentration versus the control group (0 nM) of a given time. Statistical analyses consisted of Kruskal–Wallis one-way analysis of variance on ranks, followed by multiple comparisons versus control group (Dunnett's method); ^*∗*^*p* ≤ 0.05; ^*∗∗*^*p* ≤ 0.005. (c) Representative images of inmmunofluorescence assays of MDCK monolayers, showing the expression (green) of either claudin-2 (top row) or claudin-4 (bottom row) under three experimental conditions, either control, or treated with marinobufagenin or digoxin (100 nM) for 48 hours. (d) On the left side, a representative Western blot assay, comparing the expression of claudin-4 and actin, of lysates of MDCK monolayers at four experimental conditions, either untreated (control) at zero or after 48 hours of treatment, or after 48 hours of treatment with digoxin or marinofugfagenin 100 nM. The right part compares the mean values of standardized relative density under the four experimental conditions aforementioned.

Tight junctions, perhaps the most important type of cell-cell junction of epithelia, are a network of distinct types of proteins from which claudins and occludins are major components. These structures seal adjacent cells in a narrow band just beneath their apical surface, forming a barrier that limits the flux of molecules from one compartment to another that a given epithelia separate [44, 45]. Thus, the degree of sealing is an important feature of this type of tissue and depends on the type and amount of proteins conforming the tight junction which, therefore, may be subjected of regulation by multiple factors, among which endogenous cardiac glycosides may be viable candidates. To determine whether if digoxin or marinobufagenin influences the degree of sealing between the cells, produced by tight junctions, we made measurements of Transepithelial Electrical Resistance (TER) in monolayers of MDCK cells that were treated separately with either cardiac glycoside, at the same concentrations used for GJIC assays (0.1, 1, 10, 100, and 500 nM). For this purpose, MDCK cells were seeded at confluency over semipermeable Transwell™ inserts, as described in methods, and incubated for 24 hours to mature. Then, monolayers were starved for 24 hours before adding treatments. TER measurements were taken at 0, 24, 48, and 72 hours after addition of treatments.  Figure 2 shows the relationship of TER versus time of each one of the different concentrations tested, both of digoxin (Figure 2(a)) and marinobufagenin (Figure 2(b)). To analyze the results for each compound at each time of measurement (0, 24, 48 or 72 hours), the influence of each concentration was statistically tested making multiple comparison tests (ANOVA) followed by paired comparison tests of each concentration with the control (Duncan). As can be seen in these figures, both compounds produced a remarkable effect on the magnitude as in the kinetics of TER. Mean values of untreated monolayers remained practically unchanged over time. In contrast, those from treated ones did change notoriously, depending on the concentration of the CG as well as the time of treatment. Digoxin at 100 nm and 500 nm induced a statistically significant increment of TER, but lower concentrations failed to induce this change, whereas 100 nM digoxin produced an increment of TER that was evident from 48 hours of treatment, 500 nM accelerated this change, so it was evident from 24 hours. Marinobufagenin, on the other hand, also induced a statistically significant increment of TER that was produced by lower concentrations that digoxin (from 1 nm at 24 hours). It is also noteworthy that marinobufagenin produced a maximum effect at 24 hours of treatment, which remained stable thereafter up to 72 hours.

Next, we sought to determine whether this effect on TER could be accounted for changes of expression or localization of claudins, which are major determinants of Tight Junction permeability [46]. Thus, we made immonofluorecence assays to compare the expression of claudins 2 and 4 in monolayers treated, for 48 hours, with or without digoxin or marinobufagenin in a single concentration that has been probed to produce a significant increment in TER (100 nM). As Figure 2(c) (*lower*) shows, these treatments rendered a notorious enhancement of claudin-4 expression, as compared to untreated cells, both in lateral border as well as on the cytoplasmic zone. The changes were not observed, nonetheless on the expression of claudin 2 (Figure 2(c), *upper*), indicating that cardiac glycosides promote an increment in TER by modifying the expression and localization of claudin-4 but not claudin-2. To further confirm this observation, we made western blot assays on lysates obtained from monolayers treated with or without digoxin or marinobufagenin for 48 hours and compared the level of expression by densitometric analysis, standardized with actin, of bands revealed after incubation of membranes with an antibody designed to recognize claudin-4, of samples treated versus nontreated. As Figure 2(d) shows, the study indicates a statistically significant increment in the amount of expression of claudin-4 as compared to control at 48 hours of treatment. These results, taken together lead us to conclude that both ECS, digoxin and marinobufagenin are able to influence the properties of Tight Junctions.

### 3.3. Involvement of Na-K-ATPase in a the Effect of Marinobufagenin and Digoxin on Gap Junctional Intercellular Communication and Transepithelial Tightness

As previously mentioned, Na-K-ATPase has been shown to be the receptor that transduces the action of cardiac glycosides in various physiological processes, and we have previously demonstrated that this also happens in the changes that ouabain induces on GJIC and TER of MDCK cells [36–38]. Therefore, in this work, we sought to determine whether Na-K-ATPase also participates as a receptor in the action of digoxin and marinobufagenin. For this purpose, we evaluated again the effect of both compounds on GJIC and TER, but this time on MDCK-R, a subclone highly resistant to ouabain (Kd > 4 mM), that was established by chemical mutagenesis induced by ethyl methanesulfonate [47]. It has been further shown that the acquired insensitivity to ouabain and cardiac glycosides is due to point mutations in the first transmembrane segment of the Na,K-ATPase alpha1 subunit, a domain that is also the binding site of cardiac glycosides [48].

**Figure 3:**
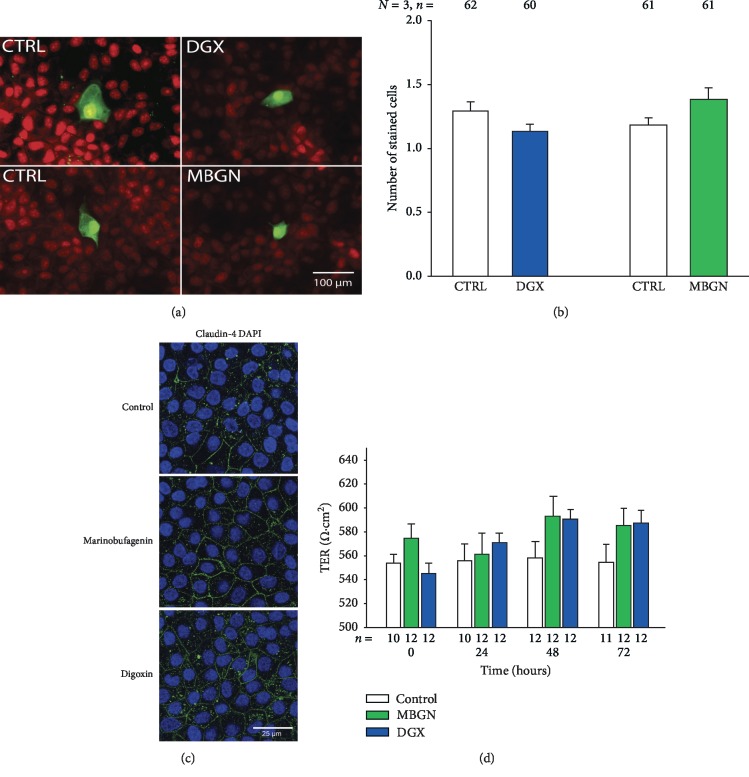
Involvement of Na-K-ATPase in the effect of marinobufagenin and digoxin on Gap Junctional Intercellular Communication (GJIC) and transepithelial electrical resistance (TER). (a) Representative images of cells stained with Lucifer Yellow, obtained after injection of a single cell, in mononolayers of MDCK-R cells, either untreated (CTRL) or treated with marinobufagenin (MBGN) 100 nM or digoxin (DGX) 10 nM during 1 hour before injections. (b) Bar chart showing the corresponding average ± SE number of cells stained in each trial. A paired comparison (*t*-student) of treatment versus its control produced no significant difference for neither ECS. (c) Representative images of immunofluorescence assays obtained on monolayers of MDCK-R, either untreated (control) or treated with digoxin or marinobufagenin 100 nM for 48 hours. Sampled were incubated with anti-claudin-4, and their expression is revealed by streptavidin FITC (green); samples were counterstained with DAPI to reveal the presence of nuclei (blue). (d) Bar chart comparing the average ± SE value of TER obtained from 9 repeats of 3 independent experiments from control and treated MDCK-R monolayers at distinct times after addition of treatment, either marinobufagenin (MBGN) 10 nM or digoxin (DGX) 100 nM. Multiple comparison statistical test (Kruskal–Wallis one-way analysis of variance on ranks) does not indicate a statistically significant difference of any treatment in any of the times in which TER was measured.

We tested the effects of digoxin and marinobufagenin on GJIC and TER, in the same way as described before, but this time only in a single concentration that had given maximum effect in the MDCK-Wild that, as shown before, was 100 and 10 nM, respectively. As shown in Figures 3(a) and 3(b), we found that neither marinofufagenin nor digoxin produced a statistically significant change on GJIC as compared to control. Similarily, TER measurements of monolayers of MDCK-R cells, treated with digoxin or marinobufagenin, were not statistically distinct from those made on untreated (control) monolayers (Figure 3(d)). Similarly, immunohistochemistry assays show no conspicuous difference in the expression of claudin-4 of monolayers treated with either digoxin or marinobufagenin as compared to those untreated (Figure 3(c)). Therefore, these results lead us to suggest that Na-K-ATPase is the primary receptor that transduces the presence of either digoxin or marinobufagenin, activating a signaling cascade pathway that leads to the effects described on GJIC or TER.

### 3.4. Involvement of c-Src and ERK1/2 in GJIC and TER Changes Induced by Digoxin and Marinobufagenin

As mentioned before, it has been described that in addition to its role as an electrogenic pump, Na-K-ATPase also acts as a signal transducer, coupled to a signalosome, that activates a number of intracellular signaling pathways [49, 50]. In a variety of cases, the signalosome has been shown to include c-Src and IP3-Receptor, both of which activate ERK1/2 and stimulate Ca2+ waves [51–54]; this signaling mechanisms, in turn, activate diverse cellular processes such as cell growth [55], apoptosis [56], and cell motility [57].

We have previously demonstrated that both c-Src and ERK1/2 participate in the signaling pathways by which the binding of ouabain to the Na-K-ATPase cause changes in several physiological properties of epithelial cells related to cell-cell contact, including Tight Junction, GJIC, cilliogenesis, and Adherens Junction [36–39].

Given this results, we considered the possibility that c-Src and ERK1/2 are components participating in the signaling pathway that produce changes in GJIC and TER upon binding of digoxin and marinobufagenin to Na-K-ATPase. For this end, we made the same experimental procedures already described, in order to compare the effect that either glycosides cause in GJIC and TER by themselves with those obtained when cells were treated with specific inhibitors of c-Src and ERK1/2. Our reasoning was that if those components are indeed involved, the presence of a specific inhibitor would suppress the effect produced by digoxin and/or marinobufagenin on GJIC and TER.

**Figure 4:**
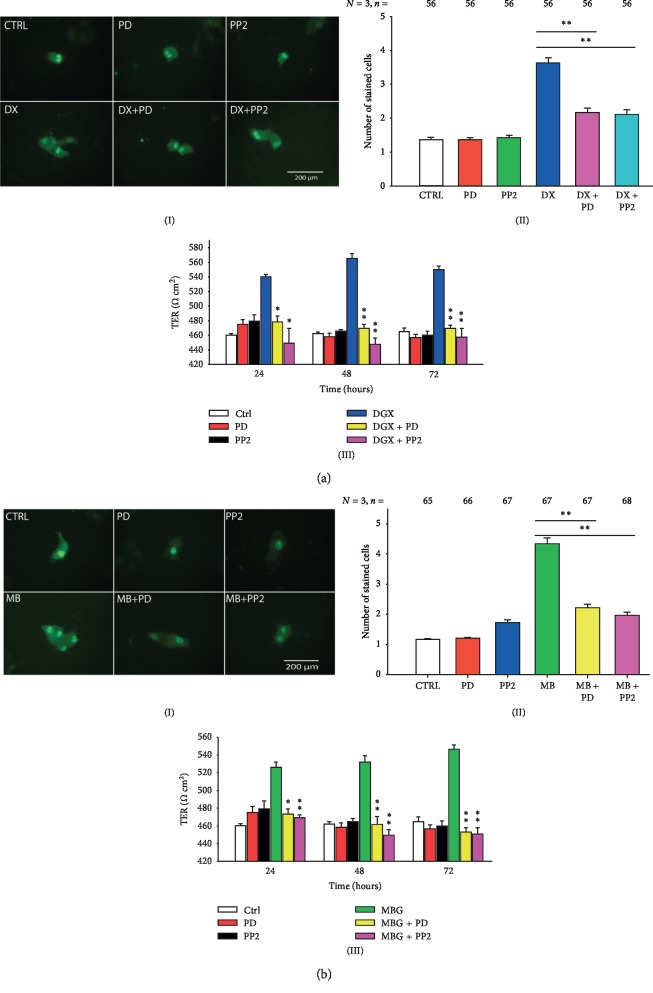
Participation of c-Src and ERK1/2 in the signaling pathway that leads to GJIC and TER increase by treatment with digoxin and marinobufagenin. (a, b) Pretreatment of monolayers of MDCK-W with inhibitors of c-Src (PP2, 10 *μ*M) and ERK1/2 (PD98059, 25 *μ*M) suppresses the enhancement of GJIC (a(I, II) and b(I, II)) and TER (a(III) and b(III)) induced by digoxin (a) or marinobufagenin (b) in MDCK cells in mature monolayers. (I, II) Effect of inhibitors on GJIC. (I) Series of representative images of dye transfer trial of each treatment assayed. (II) A bar chart comparing the average number of cells stained on each condition, as indicated below by each bar. The number of repeats is indicated in the upper part of each bar. Simple, paired comparisons (Student's *t*) were made between treatment conditions, as indicated by the lines above bars. ^*∗*^*p* ≤ 0.05; ^*∗∗*^*p* ≤ 0.001. (III) Bar charts comparing the mean ± SE (*n* = 9). Value of TER (Ohms·cm^2^) at different treatment conditions, as indicated in the inset, at 24, 48, and 72 hours of treatment ^*∗*^*p* ≤ 0.05; ^*∗∗*^*p* ≤ 0.005 of simple, pairwise comparisons (Student's *t*) of TER values obtained from monolayers treated with digoxin or marinobufagenin in the presence or absence of PD or PP2.

To test the involvement of c-Src, we analyzed the effect of PP2, a compound that has been widely demonstrated to be a potent and highly selective Src family-tyrosine kinase inhibitor [58–60]. To test the participation of ERK1/2, we assayed the effect of PD98059, a compound that has been demonstrated to be a potent (IC50 = 4 *μ*M), highly selective and cellpermeable inhibitor of MEK1 and MEK2 [61–65]. The results obtained from these assays, are summarized in Figure 4 in two parts: part (a) describes the effect of both inhibitors on the enhancement of GJIC and TER caused by digoxin and part (b) describes the corresponding effects caused by marinobufagenin. Images (A) and (B) in Figure 4(a) show that pretreatment of MDCK monolayers with PP2 (10 *μ*M for 1 hour) suppressed significantly (*p* < 0.005) the enhancement of GJIC induced by digoxin 100 nM (*light versus dark blue bars of (B) in*Figure 4(a)). Similarly, pretreatment of cells with 25 *μ*M (1 hour) PD98059, significantly (*p* < 0.005) suppressed the effect of digoxin on GJIC (*pink versus dark blue bars of (B) in*Figure 4(a)). Images (A) and (B) in Figure 4(b) describe the effect of the same inhibitors on the enhancement of GJIC induced by marinobufagenin 10 nM. As chart bar shows ((B) in Figure 4(b)) both D98059 and PP2 reduced significantly (*p* < 0.005) the enhancement of GJIC induced by marinobufagenin (*yellow and pink versus green bars*). On the other hand, image (C) in Figures 4(a) and 4(b) shows the results of TER measurements with and without the presence of f digoxin ((C) in Figure 4(a)) and marinobufagenin ((C) in Figure 4(b)). Image (C) in Figure 4(a) shows that, at all times tested, both PP2 and D98059 suppressed significantly the enhancement of TER produced by treatment with digoxin (*yellow and pink versus blue bars*). Image (C) in Figure 4(b) shows similar results from monolayers treated with marinobufagenin (*yellow and pink versus green bars*). Therefore, these results altogether, lead us to suggest that the presence of either digoxin or marinobufagenin triggers the signaling pathways that includes c-Src and ERK1/2, to produce an increase of GJIC and TER.

### 3.5. Involvement of the Rho-ROCK Pathway in the Changes of GJIC and TER Induced by Digoxin and Marinobufagenin

Next, we evaluated the possibility that in the signaling pathways that lead to the effects described, Rho proteins were involved. Rho is a family of small GTPases that participate in a wide variety of cellular functions such as vesicular trafficking, the cell cycle, transcriptomal dynamics [66], and cell polarity [67], as well as in the organization and modulation of cell junctions by the cytoskeleton [68, 69].

One of the downstream effectors of Rho A is the Rho-associated protein kinase (ROCK), a serine-threonine kinase that has been reported to be involved in the maintenance of TJ integrity in endothelial cells [70]. Several reports have described that the Rho/ROCK signaling pathway mediates the effects produced by cardiac glycosides; for instance, it has been observed that ouabain induces Rho-dependent ROCK activation, that causes membrane blebbing and apoptosis as well as hypertension [71, 72].

**Figure 5:**
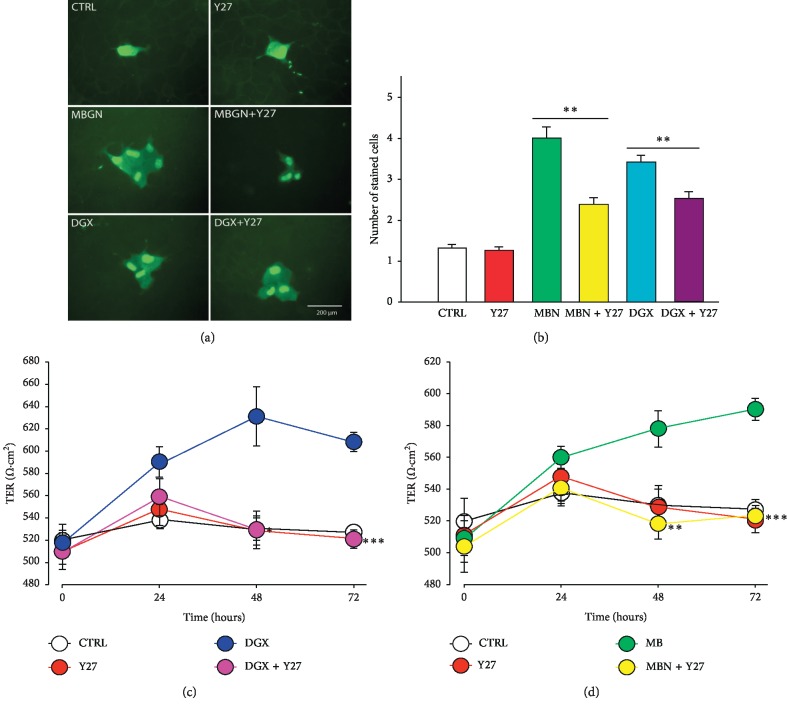
Participation of rho/ROCK in the signaling pathway that leads to GJIC and TER increase by treatment with endogenous casdiac steroids (ECS) digoxin and marinobufagenin. (a, b) Inhibition of Rho-associated protein kinase (ROCK) by pretreatment of MDCK monolayers with Y-27632 suppresses the enhancement of GJIC induced by marinobufagenin (MBGN) or digoxin (DGX). (a) Representative images of Lucifer Yellow injection trials of cells in monolayers treated with either digoxin or marinobufagenin alone or with pretreatment with Y-27632. (b) A bar chart comparing the average number of cells stained from a number of trials of each treatment as indicated in the base of bars. Simple, paired comparisons (Student's *t*) where made as indicated by bars. ^*∗*^*p* ≤ 0.05; ^*∗∗*^*p* ≤ 0.001. (c and d) Inhibition of Rho-associated protein kinase (ROCK) by pretreatment of MDCK monolayers with Y-27632 suppresses the increment of TER induced by marinobufagenin (c) or digoxin (d). (c, d) Relationship of TER versus time of the different treatment combinations indicated in the inset. Each dot represents the average ± SE value of TER (Ohms·cm^2^) of nine repeats of 3 independent assays. Simple, paired comparisons (Student's *t*) were made between ECS alone or ECS plus Y27 at each time. ^*∗*^*p* ≤ 0.05; ^*∗∗*^*p* ≤ 0.005; ^*∗∗∗*^*p* ≤ 0.001.

For this purpose, we made dye transfer assays and TER measurement in order to compare the effect that either glycosides produce by themselves with those obtained when monolayers are pretreated, with 1 *μ*M Y-27632 (Y27), a cell-permeable, highly potent and selective inhibitor of ROCK, and a Rho-associated protein kinase [73]. The statistical analysis of these results, shown in Figure 5, indicate that in fact, pretreatement of monolayers with Y-27632, significantly suppresses the enhancement of GJIC, as well as the increase of TER induced by incubation of MDCK monolayers both with digoxin and marinobufagenin. Therefore, this leads us to suggest that the Rho/ROCK signaling pathway in involved in the changes of GJIC and TER induced by digoxin and marinobufagenin.

## 4. Discussion

Cardiac glycosides have been used since thousands of years ago, first for warfare and hunting and later for therapeutical purposes, mainly to alleviate heart illness. The discovery that some of them are produced endogenously, as led to consider them as a new class of hormones, and has prompted interest to determine what their physiological role is.

Over the last few years we have turned our attention to study the influence of endogenously expressed cardiac glycosides (ECG) on the physiology of epithelial cells. Here, we have studied the influence of digoxin and marinobufagenin, at nanomolar concentrations on cell-cell communication and contact by analyzing their effect on Gap Junctional intercellular communication GJIC and the tightness of sealing between cells of which Tight Junctions are mostly responsible.

To evaluate the influence of ECG on GCIJ, we resorted to dye transfer assays, using as an estimate of GJIC the average number of cells stained as a consequence of single cell injections to evaluate the effect that treatment for 1 hour of either ECG in a nanomolare range, (from 0.1 to 500 nM) produce on this variable. As described above, we found that although both ECG induced enhancement of GJIC, they did it with distinct sensibility, as significant changes were observed from 100 nM digoxin, whereas marinobufagenin did it at lower concentrations (from 1 nM). These results are somehow similar to those obtained with ouabain [33], which at 10 nM produced a significant increment of GJIC from 15 minutes of treatment, although a maximum effect was observed after 1 hour of treatment; for that reason, we chose this time in the present work. Also is work mention that we had demonstrated by silencing assays, that conexins 32 and 43 are involved in the making of conexins mediating GJIC of MDCK [37], although synthesis of new subunits is not needed for this response within the time of treatment of 1 hour, as we found that treatment with Actinomycin D or cycloheximide, inhibitors of synthesis of new RNA and peptide subunits, did not produce a change in the effect of ouabain, along with the fact that densitometric analysis of WB of conexins 32 and 43 did not differ significantly as compared to control in those treatment conditions. Further analysis of immunofluorescence assays indicated relocalization of already synthesized units. Those findings had led us to think that the enhancement of GJIC induced by treatment of ouabain, and in this case of digoxin and marinobufagenin, could be due to a change in the kinetic properties of the conexons involved rather than the amount of them expressed, but that is a hypothesis that we pretend to pursue in a separate work. By now, it is sufficing to mention that we do not expect the expression or localization of conexins to follow the enhancement in GJIC.

On the other hand, we evaluate the influence of digoxin and marinobufagenin on degree of sealing of the tight junctions, using as a main criterion measurements of Transepithelial Electrical Resistance (TER). As described above, both ECG produced enhancement in TER from 24 hours of treatment, and this effect persisted during the time assayed (up to 72 hours). Noteworthy, here again, we found that marinobufagenin induced those changes at lower concentrations than digoxin, suggesting that both ECG evoke the same mechanisms although with distinct affinity. We also made immunohistochemical assays to probe whether the enhancement of TER correlates with changes on either the localization or the expression of claudins. We found a notorious change in the location and expression of claudin-4, but not of claudin 2. This finding was further demonstrated by western blot analysis. The increment in the amount of claudin-4 found by densitometric analysis of western blot may not be suffice to account for the increment observed in TER, which may imply that in addition to claudin-4, other claudins are contributing to the making of Tight Junction permeability, a work that could be explored more thoroughly in the future.

Furthermore, the lack of response observed in MDCK-R, a subclone that has a mutated alfa1 subunit of Na-K-ATPase, which renders a lower sensibility to cardiac glycosides (from Kd of 1 × 10^−7^ to 4 × 10^−3^ M), leads us to suggest that Na-K-ATPase, acts as a receptor that upon binding of marinobufagenin or digoxin, activates one or more signaling pathways that include cSrc and ERK1/2, as well as one or more Rho/ROCK components, which may be downstream of ERK1/2 [74]. It is also possible that Rho/ROCK directly interacts with structural components of cell junctions such as Z01 [75] and the junctional complex E-cadherin/cathenin/TEM4 [76].

**Figure 6:**
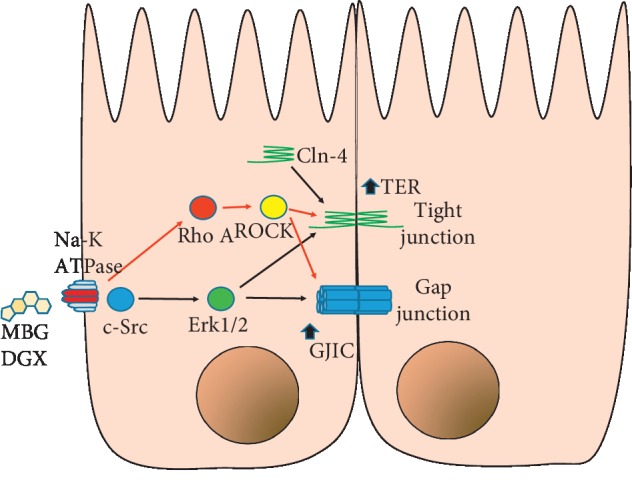
Schematic representation of the effects of ECS (marinofugagenin or digoxin). The binding of ECS to Na-K-ATPase promotes the activation of c-Src, which in turn activates ERK1/2, which promotes the activation of rho/ROCK. This in turn increases TER and CIGJ.

This description is illustrated in Figure 6, which summarizes our findings. It is worth noticing that, our results so far, with respect to the action that the ECS cause on the properties of the epithelia indicate that endogenous cardiotonics use the same receptor, and the same signaling pathways to modulate structures as different as tight junctions and gap junctions. This contrasts with other studies, which describe that the effects of ECS are often different, even antagonistic. For instance, digoxin and ouabain increases the constriction of endothelial cells from uterus but marinobufagenin does not [77]. Ouabain produces cell death of C7-MDCK cells while marinobufagenin does not [78]. Both ouabain and marinobufagenin increase blood pressure as well as smooth muscle contractility, whereas marinobufagenin has no effect [79, 80]. Strikingly, treatment with digoxin prior to ouabain abolishes the hypertensive effects of ouabain [81]. In this respect, we should consider that it is common to observe that chemical compounds exert different actions in different scenarios. Therefore, it is not surprising that a set of compounds, as ECS, exert an antagonistic action in an aspect, as hypertension and similar, or even synergistic in another, as it is in the case of their influence on epithelial renal cells.

On the other hand, we found that, although both ECS and marinobufagenin and digoxin affect GJIC and TER, they do it with distinct sensitivity. Both ECS increased TER and GJIC, marinobufagenin had a significant effect at lower concentrations than digoxin. Marinobufagenin increased TER from a 1 nM concentration (1–500 nm), while digoxin had a significant effect only at 100 and 500 nM concentrations. Similarly, marinobufagenin had a significant effect on GJIC form 0.1 nM, while digoxin did from 1 nM. In this regard, it is worth considering that there is a wide diversity of Na-K-ATPases, resulting from the association of different molecular forms of the alpha (alpha1, alpha2, alpha3, and alpha4) and beta (beta1, beta2, and beta3) subunits that constitute this molecular component [82]. Tissues in different organs express a varying proportion of subunits of the Na-K-ATPase (*α*1, *α*2, *α*3, *α*4), i.e., heterogeneity starts at molecular level. *α*1 is a ubiquitous isoform expressed practically in al cell types and is the predominant *α* isoform in epithelial tissues [83]. *α*2 is mainly expressed in the heart, skeletal muscle astrocytes, and glial cells [84]. *α*3 is present mainly at neurons and in the heart [85], and *α*4 is exclusive of sperm cells [86].

All of these *α* subunits have different degrees of affinity for cardiotonic steroids, providing the cells of different tissues with distinct sensibility to ECS. Digoxin has a greater selectivity for subunits alpha 2 and alpha 3 [87, 88], whereas marinobufagenin and ouabain have a greater selectivity for the other isoforms [23]. Therefore, it would be expected that MDCK cells, expressing only the *α*1 isoform [89], have different sensibility to DGX and marinobufagenin. Consequently, we suggest that endogenous marinobufagenin may have an important role in regulation of Tight and Gap Junctions at concentrations in the nanomolar range, while the role of digoxin on these structures may be important mainly on pathological conditions.

In a broader context, the results we show in this work support our general hypothesis that a possible role of ECS is to accentuate the epithelial phenotype, because it increases properties that are crucial in epithelial tissues. Given that we have worked with epithelia of renal origin, we could not say that the effects of ECS described here, apply to all types of tissues, not even to all types of epithelia. It is very interesting that our results seem to support the hypothesis stating that a hormonal role of ECS is to influence renal function. Since decades ago, there has been evidence suggesting that ECS functions as natriuretic agents [90, 91], although this hypothesis is still controversial [92–97]. Our findings, which have been made in epithelial cells, lead us to propose that the ECS modulates the epithelial phenotype because they influence the crucial properties of this type of tissue. However, we have not yet evaluated how these same ECS influence their transport properties, such as the expression of channels and pumps, but it is a very important aspect that we intend to pursue in a near future.

## 5. Conclusions

1. Both digoxin and marinobufagenin in concentrations in the nanomolar range induce enhancement of Gap Junctional Intercellular Communication (GJIC) and Transepithelial Electrical Resistance (TER) of MDCK cells, although they act with distinct sensibilities. Marinobufagenin induces such effects at lower concentrations that digoxin.
2. In both ECG, Na-K-ATPase is the primary receptor that transduces the presence of such compound to produce the effects on GJIC and Tight Junction sealness.
3. c-Src, ERK1/2, and Rho/ROCK are involved in the signaling pathway that produce enhancement of GJIC and TER.

## Data Availability

The data used to support the findings of this study are available from the corresponding author upon request.
